# Overexpression of m6A-factors METTL3, ALKBH5, and YTHDC1 alters HPV16 mRNA splicing

**DOI:** 10.1007/s11262-022-01889-6

**Published:** 2022-02-21

**Authors:** Xiaoxu Cui, Kersti Nilsson, Naoko Kajitani, Stefan Schwartz

**Affiliations:** 1 Department of Medical Biochemistry and Microbiology, Uppsala University, BMC-B9, 751 23 Uppsala, Sweden; 2 Department of Laboratory Medicine, Lund University, BMC-B13, 221 84 Lund, Sweden

**Keywords:** Human papillomavirus, m6A, Splicing, YTHDC1, METTL3, ALKBH5

## Abstract

We report that overexpression of the m6A-demethylase alkB homolog 5 RNA demethylase (ALKBH5) promoted production of intron retention on the human papillomavirus type 16 (HPV16) E6 mRNAs thereby promoting E6 mRNA production. ALKBH5 also altered alternative splicing of the late L1 mRNA by an exon skipping mechanism. Knock-down of ALKBH5 had the opposite effect on splicing of these HPV16 mRNAs. Overexpression of the m6A-methylase methyltransferase-like protein 3 (METLL3) induced production of intron-containing HPV16 E1 mRNAs over spliced E2 mRNAs and altered HPV16 L1 mRNA splicing in a manner opposite to ALKBH5. Overexpression of the nuclear m6A-“reader” YTH domain-containing protein 1 (YTHDC1), enhanced retention of the E6-encoding intron and promoted E6 mRNA production. We also show that HPV16 mRNAs are bound to YTHDC1 in human cells and that YTHDC1 affected splicing of HPV16 E6/E7 mRNAs produced from the episomal form of the HPV16 genome. Finally, we show that HPV16 mRNAs are m6A-methylated in tonsillar cancer cells. In summary, HPV16 mRNAs are methylated in HPV16-infected tonsillar cancer cells and overexpression of m6A-“writer” METTL3, m6A-“eraser” ALKBH5 and the m6A-“reader” YTHDC1 affected HPV16 mRNA splicing, suggesting that m6A plays an important role in the HPV16 gene expression program, at least in cancer cells.

**Supplementary Information:**

The online version contains supplementary material available at 10.1007/s11262-022-01889-6.

## Introduction

**Fig. 1:**
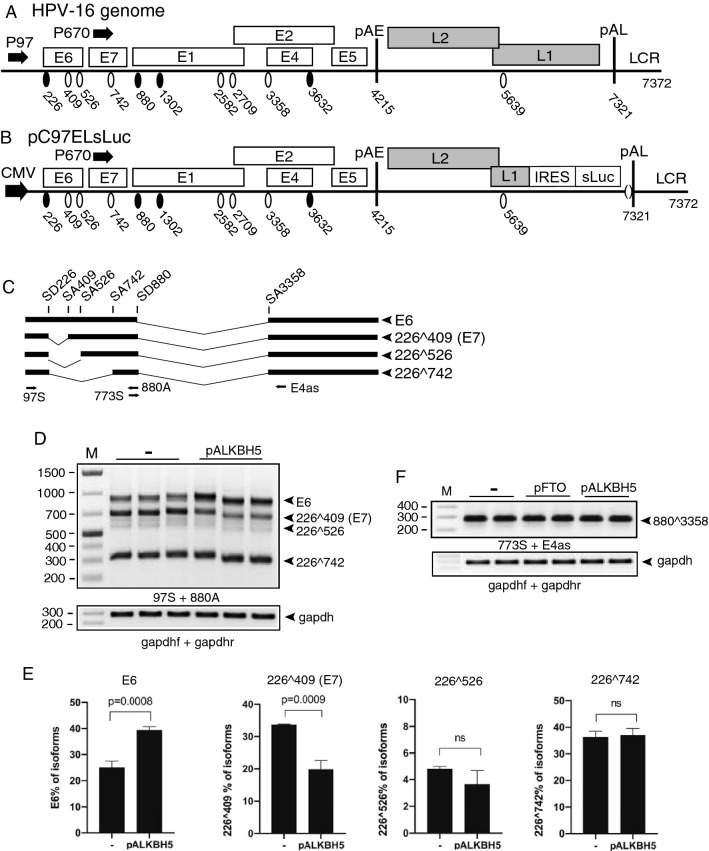
**A** Linearized HPV16 genome (numbers refer to the HPV16 reference strain GeneBank: K02718.1). Early and late genes are indicated. P97: HPV16 early promoter. P670: HPV16 late promoter. Black oval: splice donor. White oval: splice acceptor. pAE: HPV16 early polyadenylation site. pAL: HPV16 late polyadenylation site. LCR: HPV16 long control region. **B** HPV16 subgenomic plasmid pC97ELsLuc encodes all HPV16 genes. HPV16 early promoter P97 was replaced by human cytomegalovirus immediate early promoter (CMV). Secreted luciferase (sLuc) gene was integrated in the L1 gene following the poliovirus 2A internal ribosomal entry site (IRES) sequence. **C** Schematic structures of HPV16 early transcripts produced from pC97ELsLuc. Splicing at SD226 occurs independently of splicing at the downstream SD880 and generates splice variants in the E6- and E7-coding region. Arrows indicate HPV16 RT-PCR primers. **D** Effect of ALKBH5 on HPV16 E6/E7 mRNA splicing was monitored by RT-PCR with indicated primer pair on RNA extracted from triplicate transfections of pC97ELsLuc with empty pUC plasmid (−) or pALKBH5. **E** Densitometric quantitation of the results in E. The percentage of each indicated cDNA isoform of all cDNAs in a lane in the absence of ALKBH5 (−) or in the presence of ALKBH5 overexpression is displayed. **F** Effect of FTO or ALKBH5 on HPV16 E4 mRNA splicing was monitored by RT-PCR with indicated primer pair on RNA extracted from HeLa cells transfected with pC97ELsLuc and empty pUC plasmid (−), pFTO, or pALKBH5

Human Papillomaviruses (HPVs) are small DNA viruses associated with 99% of all cervical cancers, and a growing number of head and neck cancers [1, 2]. The most commonly cancer associated HPV type is HPV16 that is present in > 50% of the diagnosed HPV-caused cancers [3]. HPV16 replicates in the nucleus of keratinocytes and viral gene expression is linked to the differentiation program of the cell [4, 5]. The HPV16 genome contains two promoters (P97 and P670) and two polyadenylation signals (pAE and pAL) that separate early and late HPV genes [6] (Fig. 1A). The HPV16 E2 protein E2 regulates HPV gene expression by inhibiting the HPV early promoter and the early polyadenylation signal, thereby inducing a switch from early to late gene expression [7–9]. The pre-mRNAs produced from either HPV16 promoter are subject to RNA processing resulting in a plethora of alternatively spliced and polyadenylated mRNAs [10]. HPV16 mRNA splicing is regulated by multiple cis-acting regulatory RNA elements on the HPV16 mRNAs and their cognate cellular, trans-acting splicing factors [10–13]. HPV16 is totally dependent on the cellular splicing machinery for production of the mature mRNAs that produce HPV16 proteins.

Cellular mRNAs carry various chemical modifications that contribute to the regulation of RNA processing. m6A is believed to be the most common modification of mRNAs in eukaryotic cells. The m6A modification is reversible and is catalyzed by a methyltransferase complex consisting of methyltransferase-like-3 (METTL3), methyltransferase-like-14 (METTL14), and additional factors such as Wilms tumor 1- associated protein (WTAP), RNA-binding motif protein 15 (RBM15), KIIA1429, and zinc-finger CCCH-type 13 containing (CZ3H13) [14, 15]. Of these, METTLL3 is the catalytic subunit, while other factors such as METTL14 plays other essential roles in RNA recognition and maintenance of complex stability. Two demethylases have been identified: alkylated DNA repair protein AlkB homolog 5 (ALKBH5) and fat mass and obesity-associated protein (FTO), that both remove m6A, but with different and independent, enzymatic mechanisms [16, 17]. Thus, m6A-methylation of mRNAs is a reversible process. Taken together, it appears that m6A-methylation plays an important regulatory role in mRNA splicing.

It has been shown that m6A modifications can directly interfere with interactions between RNA and RNA-binding proteins [18, 19]. For example, secondary structures on mRNAs may be affected by the presence of m6A. Thus, m6A may affect binding of splicing factors hnRNPC and hnRNPG to their RNA target sequences [20, 21]. Proteins with an YTH-domain bind m6A-modified RNAs and affect RNA-localization, stability, polyadenylation, and translation [14, 22, 23]. The nuclear, m6A-binding YTH-protein YTHDC1 has been shown to affect splicing by specific recruitment of SR-proteins [24]. YTHDC1 also interacts with hnRNPA2 to mediate miRNA splicing [25]. Knock-down of METTL3 or deficiency in either of the demethylases ALKBH5 or FTO has been shown to affect alternative splicing of thousands of cellular mRNAs.

Since the discovery of m6A, its presence on viral RNAs has been demonstrated for a wide range of viruses, including Flaviviruses that replicate in the cytoplasm [26]. Studies aimed at unraveling the biological significance of viral RNA-methylation suggested effects on the human herpesvirus type 8 (HHV8/KSHV) lytic cycle [27], replication capacity of HIV-1 [28, 29], SV40 replication [30], and adenovirus replication and mRNA splicing [31]. HPV16 was recently shown to produce circular E6/E7 mRNAs that were m6A-methylated [32], suggesting that m6A-methylation may contribute to regulation of HPV16 gene expression at the level of RNA splicing. Since HPV-mRNAs are processed by the cellular RNA processing machinery [11], it is reasonable to speculate that m6A “writers”, “reader”, and “erasers” could affect HPV16 mRNA splicing. We therefore investigated if the m6A “writers” METTL3, METTL14 and WTAP, the m6A “erasers” ALKBH5 and FTO and the nuclear, m6A “reader” YTHDC1 could affect HPV16 mRNA splicing. We found that ALKBH5 and YTHDC1 overexpression promoted intron retention in the HPV16 early E6-coding region thereby enhancing E6 mRNA production. Furthermore, METLL3 overexpression enhanced intron retention in the HPV16 E1-coding region, thereby promoting production of E1 mRNAs over spliced E2 mRNAs. On the HPV16 late mRNAs, ALKBH5 overexpression caused skipping of the internal exon in the HPV16 E4-coding region of the HPV16 late L1 mRNAs, suggesting that m6A also control late gene expression of HPV16. We concluded that various m6A “writers”, “erasers”, and “readers” altered HPV16 mRNA splicing, suggesting an important role for these factors in the HPV16 gene expression program.

## Materials and methods

### Cells

**Fig. 2:**
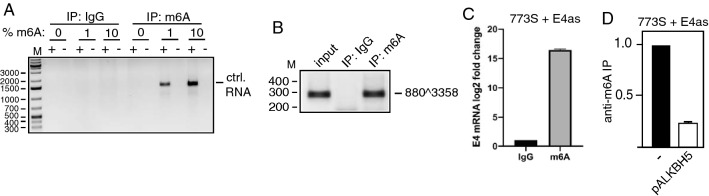
**A** Vector RNA was synthesized in vitro (Supplementary Fig. S1) in the presence of 0%, 1%, or 10% m6A-nucleotide and subjected to immunoprecipitation with either IgG or anti-m6A antibody followed by RT-PCR with T7 forward and reverse primers (Supplementary Table 1). **B** Hela cells transfected with pC97ELsLuc were subjected to CLIP assay. The RNA–protein complexes were immunoprecipitated with IgG or by anti-m6A antibody followed by RT-PCR with primers 773S and E4as on the extracted RNA. **C** Hela cells transfected with pC97ELsLuc were subjected to CLIP assay. The RNA–protein complexes were immunoprecipitated with IgG or by anti-m6A antibody followed by RT-qPCR with primers 773S and E4as on the extracted RNA. Fold difference between RT-qPCR on RNA immunoprecipitated with anti-m6A antibody over IgG is shown. **D** Hela cells transfected by pC97ELsLuc in the absence or presence of pALKBH5 were subjected to CLIP assay. The RNA–protein complexes were immunoprecipitated with IgG or anti-m6A antibody followed by RT-qPCR with primers 773S and E4as on the extracted RNA. Fold difference between RT-qPCR on RNA immunoprecipitated with anti-m6A antibody over IgG is shown

HeLa and C33A2 cells were cultured in Dulbecco’s modified Eagle medium (DMEM) (GE Healthcare Life Science HyClone Laboratories) with 10% heat-inactivated fetal bovine calf serum (GE Healthcare Life Sciences HyClone Laboratories) and penicillin/streptomycin (Gibco Thermo Fisher Science). HN26 cells were cultured in RPMI-1640 medium (GE Healthcare Life Science HyClone Laboratories) supplemented with 10% Bovine Calf Serum (GE Healthcare Life Science HyClone Laboratories), 1% Sodium Pyruvate solution (Sigma-Aldrich),1% Non-Essential Amino Acid (Sigma-Aldrich), and Gentamycin. The C33A2 cell line originates from C33A and has the subgenomic HPV16 plasmid pBELsLuc (Fig. 2A, B) stably integrated into the genome [33]. In the pBELsLuc construct, part of the L1 coding region have been exchanged for a poliovirus 2A internal ribosome entry site (IRES) in front of the Metridia longa secreted luciferase (sLuc) gene. Induction of HPV16 late gene expression results in the secretion of sLuc into the cell culture medium. The HN26 cell line has previously been described and is a tonsillar cancer cell line with episomal HPV16 DNA [34, 35]. Briefly, the HN26 cells are derived from a tumor of a 48-year-old nonsmoking man with non-keratinizing, HPV16-positive tonsil oral squamous cell carcinoma, stage T2N0M0. The HN26 cells contain episomal HPV16 DNA and have an intact p53 gene.

### Plasmids and transfections

The following plasmids have been described previously: pC97ELsLuc [36, 37], pBELsLuc [36, 37], pHPV16AN [36, 37], and pX856F [38]. PcDNA3-FLAG-HA-hYTHDC1 (#85167), pcDNA3/Flag-METTL3 (#53739), pcDNA3/Flag-METTL14 (#53740), pcDNA3/Flag-WTAP (#53741), pFRT/TO/HIS/FLAG/HA-ALKBH5 (#38073) were purchased from Addgene. pcDNA3.1^+^/C-(K)DYK-FTO was purchased from GenScript. Transfections were made with Turbofect according to the manufacturer’s protocol (Fermentas). Briefly, a mixture of 2 µl Turbofect per 1 µg DNA and 100 µl of DMEM without serum was incubated at room temperature for 25 min prior to dropwise addition to 60-mm plates with subconfluent HeLa cells.

### In vitro RNA syntheses

In vitro RNA was synthesized using the TranscriptAid T7 High Yield Transcription Kit (#K0441, Thermo Scientific). The provided control template DNA was used according to the manufacture´s instruction, the sequence of the control DNA is listed in Supplementary Fig. S1. Three reactions were made in parallel in which 0-, 1-, or 10% of the ATP-pool were substituted for the N6-methyladenosine base analogue m6A (S3190, Selleckchem).

### RNA extraction from transfections and RT-PCR

Total RNA was extracted 24 h and/or 48 h post-transfection using TRI Reagent (SIGMA Aldrich Life Science) and Direct-zol RNA MiniPrep (ZYMO Research) according to the manufacturer’s protocols. cDNA was synthesized from 500 ng RNA in a 20 μl reaction at 37 °C with M-MLV Reverse Transcriptase (Invitrogen) and random primers (Invitrogen) according to the protocol of the manufacturer. One microliter of cDNA was subjected to polymerase chain reaction (PCR) amplification.

### UV-crosslinking and immunoprecipitation with m6A antibody

C33A2 and HN26 cells were grown in 10-cm dishes until reaching 80% confluency. Total RNA was then harvested using TriReagent (Invitrogen), followed by DNase I (Thermo Scientific) treatment for 1 h at 37 °C according to manufacturer’s protocols. RNA was extracted by phenol/chloroform extraction followed by ethanol precipitation and resuspension in 20 µl H_2_O. 20 of µg RNA was then diluted in 450 µl immunoprecipitation (IP) buffer (50 mM Tris pH 7.4, 100 mM NaCl, 0.05% NP40), and 5ug of either IgG or Anti-m6A antibody (Abcam rabbit polyclonal (ab151230)) and 5 µl RI Ribolock (Thermo Scientific). In some experiments, the solutions were transferred into 3 cm cell culture dishes and crosslinked twice with 0.15 J cm^2^ UV light (254 nm) in a Stratalinker (Agilent). Samples were incubated overnight on a rotating wheel at 4 °C. Thirty microliters (0.6 mg) of Dynabeads Protein G (#10004D) (Invitrogen) were added to the antibody-RNA mixture followed by incubation for 2 h, at 4 °C. The beads were washed twice on a magnetic stand with high-salt buffer (50 mM Tris pH7.4, 1 M NaCl, 1 mM EDTA, 1% NP40, 0.1% SDS), and 4 times with IP buffer. RNA was eluted by phenol/chloroform extraction. All immunoprecipitated antibody–RNA complexes were extracted directly from the immunoprecipitations, purified, and analyzed by RT-PCR and/or qRT-PCR. RNA was dissolved in 20 µl of water and ten microliters were reverse transcribed using random primers (Invitrogen) and M-MLV reverse transcriptase for 50 min at 37 °C (Invitrogen). The remaining ten microliters served as negative controls. 1 µl cDNA was subjected to RT-PCR with primers indicated in the figures (see Supplementary Table 1 for primer sequences). For quantification of immunoprecipitated RNA, RT-qPCR was performed on 1 μl of cDNA synthesized as described above. RT-qPCR was performed in a MiniOpticon (Bio-Rad) using the Sso Advanced SYBR Green Supermix (Bio-Rad) according the manufacturers protocol. For primer sequences, see Supplementary Table 1. To calculate relative expression levels of each gene, the C_T_ values of the target gene from treated cells were normalized to the levels in DMSO treated cells.

### Western blotting

Cell extracts for Western blotting were obtained by resuspending transfected cells in radioimmunoprecipitation assay (RIPA) buffer consisting of 50 mM Tris HCl pH 7.4, 150 mM NaCl, 1% NP-40, 0.5% sodium deoxycholate, 0.1%SDS, 2 mM EDTA, 1 mM DTT, and protease inhibitor (Sigma Aldrich), followed by centrifugation at full speed for 20 min and collection of the supernatants. Proteins were denatured by boiling in Laemmli buffer. After SDS-PAGE, the proteins on the gels were transferred onto nitrocellulose membranes, blocked with 5% nonfat dry milk in PBS containing 0.1% Tween 20, and stained with specific primary antibodies (anti-Flag antibody M2 F1804 (Sigma Aldrich), anti-beta-actin antibody A5441 (Sigma Aldrich) or rabbit anti-ALKBH5 antibody #ab174124 (Abcam)) to the indicated proteins followed by incubation with secondary antibody conjugated with horseradish peroxidase and detection with chemiluminescence reagents.

### Lentiviral based shRNA for knockdown

Lentivirus for the short hairpin RNA (shRNA)-mediated knockdown of ALKBH5 was generated by co-transfection of HEK293T cells with a pLKO.1-vector or pLKO.1-vector carrying specific shRNA together with the packaging vector pMISSION-GAG-POL and a vesicular stomatitis virus G protein expressing vector pMISSION-VSV-G. pLKO.1 was purchased from Sigma-Aldrich (SHC001). Two days post-transfection, lentivirus-containing supernatants were harvested, centrifuged to remove cellular debris, and filtered with a 0.45-um filter. Lentivirus production efficiency was determined in parallel using a GFP overexpression lentivirus vector. C33A2 cells were inoculated with stocks of recombinant lentiviruses by centrifugation at 2000 g for 2 h at room temperature in the presence of 10 lg/ml polybrene (Fisher Scientific). Empty pLKO.1-vector was used as negative control. Cells were then resuspended and grown in normal RPMI media for 2 days, after which transduced cells were selected in the presence of puromycin (1 ug/ml). Cells were either harvested for Western blotting or for RNA extraction and RT-PCR.

### UV-crosslinking and immunoprecipitation (CLIP)

Transfected HeLa cells grown in 10-cm dishes were washed by ice-cold PBS followed by crosslinking twice with 0.4 J/cm^2^ UV light (254 nm) in a bio-link cross-linker (Biometra). Cytoplasmic extracts were prepared as described above. Whole cell lysates were prepared by resuspending cells in one ml of RIPA buffer and incubated on ice for 30 min with occasional vortexing to lyse cells. For immunoprecipitations, 2 µg of the anti-flag antibody (M2, Sigma Aldrich) or mouse IgG was incubated at 4 °C overnight in 0.5 ml of cell lysate. 20 µl of Dynabeads Protein G (10004D, Invitrogen) and 20 µl Dynabeads Protein A (10001D, Invitrogen) were blocked with 1% BSA for 0.5 h, washed three times in RIPA buffer, and then added to the antibody–protein mixtures followed by incubation for 1 h at 4 °C. The beads were washed three times with buffer I (50 mM Tris HCl pH 7.4, 300 mM NaCl, 0.5% NP-40, 1 mM EDTA, 1 mM DTT), three times with buffer II (50 mM Tris HCl pH 7.4, 800 mM NaCl, 0.5% NP-40, 1 mM EDTA, 1 mM DTT), and three times with buffer III (50 mM Tris HCl pH 7.4, 800 mM NaCl, 250 mM LiCl, 0.5% NP-40, 1 mM EDTA, 1 mM DTT). RNA was eluted by phenol/chloroform extraction and ethanol-precipitated and dissolved in 20 µl of water. 10 µl of immunoprecipitated RNA was reverse transcribed using M-MLV reverse transcriptase (Invitrogen) and random primers (Invitrogen) according to the protocol of the manufacturer. Two microliters of cDNA were subjected to PCR amplification using HPV16-sepcific primers.

### Quantitations

The software used to determine band intensity in Western blots and RT-PCR gels is “Image Lab 6.1.0” and quantitations were performed with the software “Prism GraphPad 8.4.0”.

## Results

### Overexpression of the m6A “eraser” ALKBH5 alters HPV16 alternative mRNA splicing

To investigate if m6A-“erasers” (ALKBH5, FTO), m6A-writers (METTL3, METTL14, WTAP) and m6A-readers (YTHDC1) affected HPV16 mRNA splicing, HPV16 reporter plasmid pC97ELsLuc (Fig. 1B) was cotransfected into HeLa cells with plasmids expressing ALKBH5, FTO, METTL3, METTL14, WTAP, or YTHDC1 (Supplementary Fig. S2A). First we analyzed the effect of FTO and ALKBH5 on alternatively spliced HPV16 E6/E7 mRNAs (Fig. 1C). The major 5’-splice site in the E6- and E7-coding region named SD226 may be spliced to either SA409, SA526, or SA742 (Fig. 1C). Splicing between HPV16 splice sites SD226 and SA409 generates the E7 mRNA, whereas the significance of SA526 and SA742 is less clear. Retention of the E6-encoding intron downstream of SD226 generates the E6-encoding mRNA. First, pC97ELsLuc was cotransfected with plasmids expressing flag-tagged ALKBH5 or FTO m6A eraser proteins (Supplementary Fig. S2A). Triplicate transfections of ALKBH5 with pC97ELsLuc (Fig. 1D) followed by quantitation revealed that the splicing inhibitory effect of ALKBH5 on the HPV16 E6/E7 mRNAs was subtle, but reproducible (Fig. 1E). Splicing from SD880 to SA3358 appeared unaffected by FTO and ALKBH5 (Fig. 1F). FTO did not affect HPV16 E6/E7 mRNA splicing (data not shown). In conclusion, overexpression of ALKBH5 inhibited production of the HPV16 mRNAs spliced between 226 and 409 and promoted retention of the E6-encodig intron thereby favoring production of E6 mRNAs over E7 mRNAs.

### Immunoprecipitation of HPV16 mRNAs by anti-m6A antibody.

Since ALKBH5 is an m6A “eraser”, we investigated if ALKBH5 overexpression reduced m6A-methylation of HPV16 mRNAs by immunoprecipitating m6A-methylated HPV16 mRNAs in the absence or presence of ALKBH5 overexpression. First we determined the specificity of the m6A antibody for m6A-methylated RNA. A cloning vector RNA sequence (Supplementary Fig. S1) was synthesized in vitro using nucleotide mixtures consisting of both adenosine and m6A. RNA sequences with mixtures containing 0%-, 1%-, or 10%-m6A nucleotides were subjected it to immunoprecipitation with Abcam anti-m6A antibody followed by RT-PCR. As can be seen, only RNA containing m6A was immunoprecipitated by the m6A antibody while none of the RNAs were immunoprecipitated by IgG under the experimental conditions used here (Fig. 2A). Next, RNA extracted from HeLa cells transfected with HPV16 reporter plasmid pC97ELsLuc and empty pUC plasmid (−) or pALKBH5 expression plasmid were subjected to immunoprecipitation with anti-m6A antibody and RT-PCR on RNA extracted after immunoprecipitation. The 773 s-E4as primer pair detects all HPV16 early mRNAs spliced between SD880 and SA3358 (Fig. 1C). The results revealed that the anti-m6A antibody efficiently immunoprecipitated HPV16 mRNAs compared to IgG, demonstrating that the HPV16 mRNAs produced by pC97ELsLuc are m6A-methylated in the transfected cells (Fig. 2B). The results were confirmed by RT-qPCR (Fig. 2C). Next we immunoprecipitated RNA with anti-m6A antibody from cells transfected with pC97ELsLuc and empty pUC plasmid or ALKBH5 expressing plasmid and performed RT-qPCR. As can be seen, overexpression of ALKBH5 reduced the amount of HPV16 mRNAs immunoprecipitated by the m6A antibody (Fig. 2D). These results demonstrated that ALKBH5 overexpression reduced the levels of m6A-methylation on HPV16 mRNAs and that this reduction correlated with retention of the E6-encoding intron on the HPV16 mRNAs. Taken together, our results indicated that a reduction of m6A-methylation of HPV16 mRNAs inhibited HPV16 mRNA splicing and promoted intron retention, suggesting that increased m6A-methylation of HPV16 mRNAs would enhance splicing of the HPV16 E6/E7 mRNAs and promoted production of HPV16 E7 mRNAs.

### Overexpression of the m6A “writer” METTL3 alters HPV16 alternative mRNA splicing

**Fig. 3:**
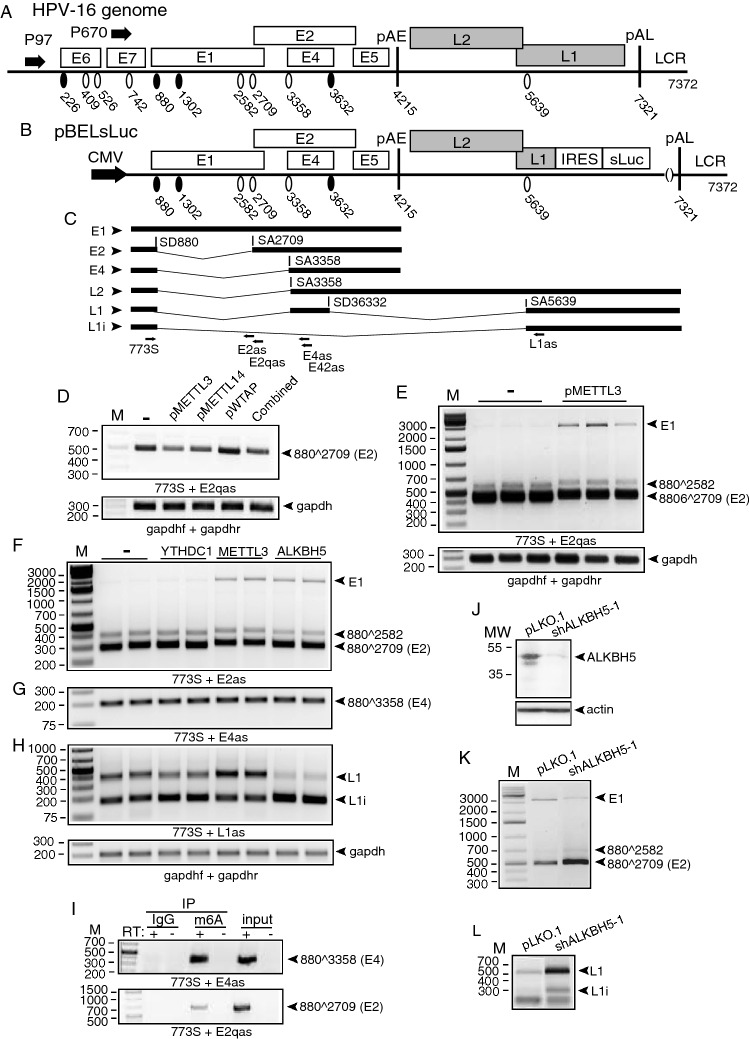
**A** Linearized HPV16 genome (numbers refer to the HPV16 reference strain GeneBank: K02718.1). Early and late genes are indicated. P97: HPV16 early promoter. P670: HPV16 late promoter. Black oval: splice donor. White oval: splice acceptor. pAE: HPV16 early polyadenylation site. pAL: HPV16 late polyadenylation site. LCR: HPV16 long control region. **B** Schematic representation of HPV16 subgenomic plasmid pBELsLuc. Transcription from pBELsLuc is driven by the human cytomegalovirus immediate early promoter (CMV). Secreted luciferase (sLuc) gene was integrated in the L1 gene following the poliovirus 2A internal ribosomal entry site (IRES) sequence. **C** Schematic structures of a subset of HPV16 alternatively spliced mRNAs produced from pBELsLuc. Arrows indicate HPV16 RT-PCR primers. **D** Effect of METTL3, METTL14, WTAP, or all three combined on HPV16 E2 mRNA splicing was monitored by RT-PCR with indicated primer pair on RNA extracted from HeLa cells transfected with pBELsLuc and pMETTL3, pMETTL14, pWTAP or pMETTL3, pMETTL14 and pWTAP combined. **D** Effect of METTL3 on HPV16 E2 mRNA splicing was monitored by RT-PCR with indicated primer pair on RNA extracted from HeLa cells transfected with pBELsLuc and empty pUC plasmid (−) or pMETTL3 in triplicates. **E** Effect of METTL3 on HPV16 E2 mRNA splicing was monitored by RT-PCR with indicated primer pair on RNA extracted from HeLa cells transfected with pBELsLuc and pUC (−) or pMETTL3 in triplicates. The RT-PCR was optimized for detection also of the larger E1 mRNAs with retained E1-encoding intron (E1). Effect of YTHDC1, METTL3, or ALKBH5 on HPV16 E2- (**F**), E4- (**G**), or L1- (**H**). mRNA splicing was monitored by RT-PCR with indicated primer pair on RNA extracted from HeLa cells transfected with pBELsLuc and empty pUC plasmid (−), pYTHDC1, pMETTL3, or pALKBH5 in duplicates. **I** RNA immunoprecipitation of RNA extracted from C33A2 cells using IgG or anti-m6A antibody followed by RNA extraction and RT-PCR with indicated primers. Input: RT-PCR on RNA extracted from C33A2 cells in the absence of immunoprecipitation. **J** Western blot with anti-ALKBH5 or actin antibody on proteins extracted from C33A2 cells infected with lentivirus pLKO.1-vector or pLKO.1 with shRNA to ALKBH5 (shAKBH5-1). **K** Effect of ALKBH5 knock-down on HPV16 E2 mRNA splicing (880^2709) was monitored by RT-PCR with RT-PCR primers 773S and E2qas on RNA extracted from C33A2 cells infected with pLKO.1-vector or pLKO.1 with shRNA to ALKBH5 (shALKBH5-1). The RT-PCR was optimized for detection also of the larger E1 mRNAs with retained E1-encoding intron (E1). **L** Effect of ALKBH5 knock-down on HPV16 L1/L1i mRNA splicing was monitored by RT-PCR with RT-PCR primers 773S and L1as on RNA extracted from C33A2 cells infected with pLKO.1-vector or pLKO.1 with shRNA to ALKBH5 (shALKBH5-1)

Next, we cotransfected HPV16 reporter plasmid pC97ELsLuc with plasmids that overexpressed the m6A-“writers” METTL3, METTL14, and WTAP (Supplementary Fig. S2A). None of these proteins appeared to significantly affect HPV16 E6/E7 mRNA splicing (Supplementary Fig. S2B) nor did they appear to affect splicing of HPV16 mRNAs between SD880 and SA3358 (data not shown). However, METTL3 overexpression exerted an inhibitory effect on HPV16 E2 mRNAs (880^2709^) produced from pC97ELsLuc (Supplementary Fig. S2C). This inhibitory effect of HPV16 E2 mRNA splicing was specific for METTL3 and was not observed with METLL14 or WTAP, the other two m6A writers analyzed here (Supplementary Fig. S2C). We decided to investigate the effect of METTL3 on E2 mRNA splicing further using HPV16 reporter plasmid pBELsLuc that lacks the E6/E7-coding region but encodes E1 and E2 (Fig. 3A, B). The pBELsLuc plasmid has the potential to produce HPV16 E1, E2, and E4 mRNAs as well as L2 and two alternatively spliced L1 mRNAs named L1i and L1 (Fig. 3C). Cotransfection of the m6A “writers” with HPV16 reporter plasmid pBELsLuc reproduced the effect on the E2 mRNAs previously observed with HPV16 reporter plasmid pC97ELsLuc: METTL3 inhibited HPV16 E2 mRNA splicing, whereas WTAP did not (Fig. 3D). However, METTL14 inhibited production of spliced E2 mRNA from pBELsLuc, but not from pC97ELsLuc (Fig. 3D). This may be explained by the fact that pBELsLuc produces relatively few alternatively spliced HPV16 mRNAs compared to pC97ELsLuc and therefore is likely to be a more sensitive reporter plasmid for E1/E2 mRNA splicing than pC97ElsLuc. In addition, METTL14 does play a significant role in RNA recognition and stability of the methyltransferase complex. Since the effect of METTL14 overexpression was HPV16-plasmid specific, we did not pursue these results further. Performing RT-PCR under conditions that allowed detection also of the larger, HPV16 E1-intron encoding mRNAs in addition to the spliced E2 mRNAs confirmed that METLL3 inhibited production of the spliced E2 mRNAs (Fig. 3E) and revealed that METTL3 promoted production of the E1-intron containing mRNAs (Fig. 3E). Unexpectedly, overexpression of ALKBH5 also inhibited E2 mRNA splicing and promoted retention of the E1-encoding intron, but to a lesser extent than METTL3 (Fig. 3F). Splicing between SD880 and SA3358 appeared unaffected by both METTL3 and ALKBH5 (Fig. 3G). Taken together, these results supported the idea that m6A-methylation modulate splicing of HPV16 early E1/E2 and E6/E7 mRNAs.

### METTL3 and ALKBH5 alter HPV16 late L1 mRNA splicing

We also monitored the effect of the m6A “writers” and “erasers” on the two alternatively spliced, HPV16 late L1 mRNAs here named L1 and L1i (Fig. 3C). Overexpression of METTL3 enhanced inclusion of the central exon on the L1 mRNAs and inhibited splicing directly from SD880 to SA5639, thereby promoting production of HPV16 L1 mRNAs over L1i mRNAs (Fig. 3H). Overexpression of ALKBH5 altered HPV16 L1 mRNA splicing by causing skipping of the central exon of the L1 mRNAs and enhancing direct splicing from SD880 to SA5639, thereby promoting production of HPV16 L1i mRNAs over L1 mRNAs (Fig. 3H). FTO overexpression did not affect splicing of the HPV16 L1 mRNAs (Supplementary Fig. S2D). To further support a role for METLL3 and ALKBH5 in the splicing of E1/2- and L1/L1i mRNAs, we attempted to knock-down these two proteins in the C33A2 cell line by using lentiviral vectors carrying shRNA to either METLL3 or ALKBH5. The C33A2 cell line carries the HPV16 reporter plasmid pBELsLuc stably integrated into its genome (Fig. 3B) and constitutively express the depicted HPV16 E1 and E2 mRNAs (Fig. 3C). We first confirmed that HPV16 mRNA are m6A-methylated in the C33A2 cell line by immunoprecipitation of the HPV16 mRNAs by anti-m6A antibody. The results revealed that anti-m6A antibody, but not IgG, immunoprecipitated HPV16 mRNAs extracted from C33A2 cells, indicating that they were m6A-methylated (Fig. 3I). We were unable to reproducibly knock-down METTL3, but ALKBH5 was efficiently knocked down after infection with a lentivirus carrying shRNA to ALKBH5 (Fig. 3J). Knock-down of ALKBH5 caused an increase in E2 mRNA splicing (Fig. 3K) and enhanced inclusion of the central exon on the L1 mRNAs (Fig. 3L). As expected, knock-down of ALKBH5 had the opposite effect of ALKBH5 overexpression on the splicing of E1/E2 mRNAs (compare Fig. 3F and K) and L1/L1i mRNAs (compare Fig. 3H and L). We concluded that the m6A “eraser” ALKBH5 and the m6A “writer” METTL3 affected alternative splicing of HPV16 early and late mRNAs, further supporting the idea that m6A plays an important role in the control of HPV16 alternative mRNA splicing and gene expression.

### Overexpression of the m6A “reader” YTHDC1 inhibits HPV16 E6/E7 mRNA splicing, thereby promoting production of full-length E6 mRNAs

**Fig. 4:**
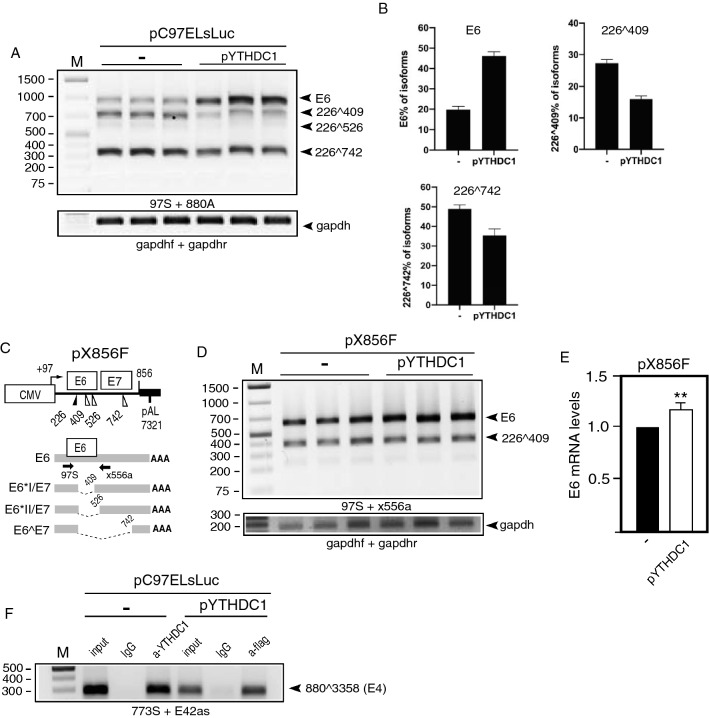
**A** RT-PCR with primers 97S and 880A on RNA extracted from HeLa cells transfected in triplicates with pC97ELsLuc and empty pUC plasmid (−) or pYTHDC1 plasmid. **B** Densitometric quantitation of gel image in (A). The percentage of each indicated cDNA isoform of all cDNAs in a lane in the absence of YTHDC1 (−) or in the presence of YTHDC1 overexpression is displayed. **C** Schematic representation of the HPV16 E6- and E7-encoding reporter plasmid pX856F. The human cytolomegalovirus promoter (CMV), the HPV16 E6 and E7 open reading frames, the HPV16 polyadenylation signal (pAL), and splice sites (226, 409, 526, and 742) are indicated. Alternatively spliced mRNAs produced by pX856F are displayed. Arrows represent RT-PCR primers 97S and x556a. **D** RT-PCR with primers 97S and x556a on RNA extracted from HeLa cells transfected in triplicates with pX856F and empty pUC plasmid (−) or pYTHDC1 plasmid. **E** Densitometric quantitation of gel image in (**D**). **F** CLIP assay was performed on cell extracts from HeLa cells transfected with pC97ELsLuc and empty pUC plasmid (−) or pYTHDC1 that produces flag-tagged YTHDC1. Immunoprecipitation of RNA–protein complexes was performed with anti-YTHDC1 antibody (a-YTHDC1) or with anti-flag antibody (a-flag). RT-PCR was performed with HPV16 primers 773S and E42as. For location of RT-PCR primers see Fig. 3C

Since overexpression of the m6A “writers” and “erasers” METTL3 and ALKBH5 affected alternative splicing of various HPV16 mRNAs, one may speculate that a nuclear “reader” of m6A-containing mRNAs might alter HPV16 mRNA splicing as well. The most well known nuclear m6A “reader” is YTHDC1. Plasmid pYTHDC1 produces flag-tagged YTHDC1 (Supplementary Fig. S2A) and was co-transfected with HPV16 reporter plasmid pC97ELsLuc (Fig. 1B). Overexpression of YTHDC1 altered HPV16 E6/E7 mRNA splicing (Fig. 4A), but did not affect HPV16 mRNAs spliced from SD880 to SA2709 (E2) (Fig. 3F), SD880 to SA3358 (E4) (Fig. 3G), or L1/L1i mRNA splicing (Fig. 3H). Quantitation of the effect of YTHDC1 on E6/E7 mRNA splicing in triplicate transfections (Fig. 4B) demonstrated that YTHDC1 primarily enhanced intron retention to promote production of E6-encoding mRNAs, and reduced production of spliced HPV16 mRNAs (226^409) (Fig. 4B). Next we investigated if YTHDC1 acted directly on the E6/E7-coding region by co-transfecting YTHDC1 in triplicates with the smaller HPV16 reporter plasmid pX856F [38] that encodes only E6 and E7 (Fig. 4C). Overexpression of YTHDC1 promoted retention of the E6-encoding exon at the expense of the E7 mRNA spliced from SD226 to SA409 (Fig. 4D, E). These results indicated that YTHDC1 acted directly on the E6/E7-coding region. To investigate if YTHDC1 binds directly to HPV16 mRNAs, we performed a CLIP assay on YTHDC1 on HeLa cells transfected with pC97ELsLuc, either in the presence of empty pUC plasmid (−) or pYTHDC1 that produced flag-tagged YTHDC1. The results revealed that anti-YTHDC1 antibody immunoprecipitated HPV16 mRNAs from pC97ELsLuc and pUC transfected cells (Fig. 4F) and that anti-flag antibody immunoprecipitated HPV16 mRNAs from cells cotransfected with pC97ELsLuc and flag-tagged pYTHDC1 (Fig. 4F). Thus both endogenous and overexpressed YTHDC1 interacted with HPV16 mRNAs in the transfected HeLa cells.

### YTHDC1 inhibits splicing of HPV16 mRNAs produced from episomal HPV16 genome

**Fig. 5:**
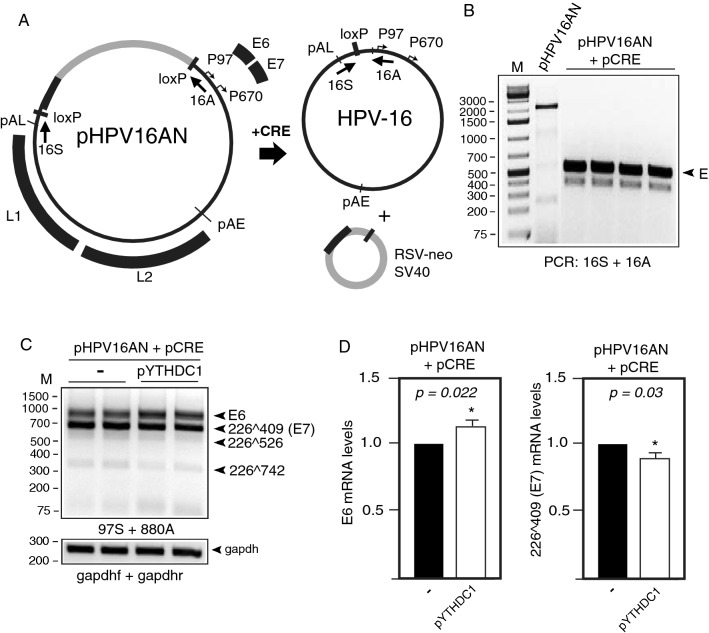
**A** Schematic representation of the pHPV16AN plasmid that carries the full-length HPV16 genome flanked by loxP sites. Co-transfection of pHPV16AN and pCRE results in excision and circulation of the viral DNA at the loxP sites to generate episomal HPV16 DNA. Positions of PCR primers 16A and 16S used to monitor HPV16 genome excision and circularization are indicated. **B** Gel shows PCR with primers 16A and 16S on DNA harvested 48 h post-transfection. E, episomal HPV16 DNA. **C** RT-PCR on RNA extracted from HeLa cells transfected with pHPV16AN and pCRE with empty pUC plasmid (−) or pYTHDC1. Transfections were performed in duplicates. RT-PCR was performed with the indicated RT-PCR primers. **D** Densitometric quantitation of gel images of RT-PCR products shown in (**C**). The RT-PCR band representing E6 mRNAs as well as the band representing spliced (226^409^) E7 mRNAs were quantified and fold difference in the absence or presence of YTHDC1 is shown in the graph. *p*-values are indicated

Next, we analyzed the effect of YTHDC1 on the full-length HPV16 genome by using plasmid pHPV16AN (Fig. 5A). This full-length HPV16 genome is flanked by loxP sites and co-transfection with a cre-expressing plasmid releases the episomal form of the HPV16 genome (Fig. 5A). The cre/lox reaction is controlled with PCR with primers 16S and 16A that discriminate between the transfected pHPV16AN plasmid DNA and the cre/loxed HPV16 episomal DNA (Fig. 5B). Analyses of the E6/E7 mRNAs produced by the episomal form of the HPV16 genome in the absence or presence of overexpressed YTHDC1 revealed that YTHDC1 inhibited E6/E7 mRNA splicing and promoted retention of the E6-encoding intron at the expense of the spliced E7 mRNAs (226^409^) (Fig. 5C). However, quantitation revealed that the significance was relatively low (*p* < 0.05) (Fig. 5D). We concluded that YTHDC1 enhanced production of E6 mRNAs by inhibiting HPV16 mRNA splicing.

### HPV16 mRNAs are m6A-methylated in HPV16-infected tonsillar cancer cell line HN26

**Fig. 6:**
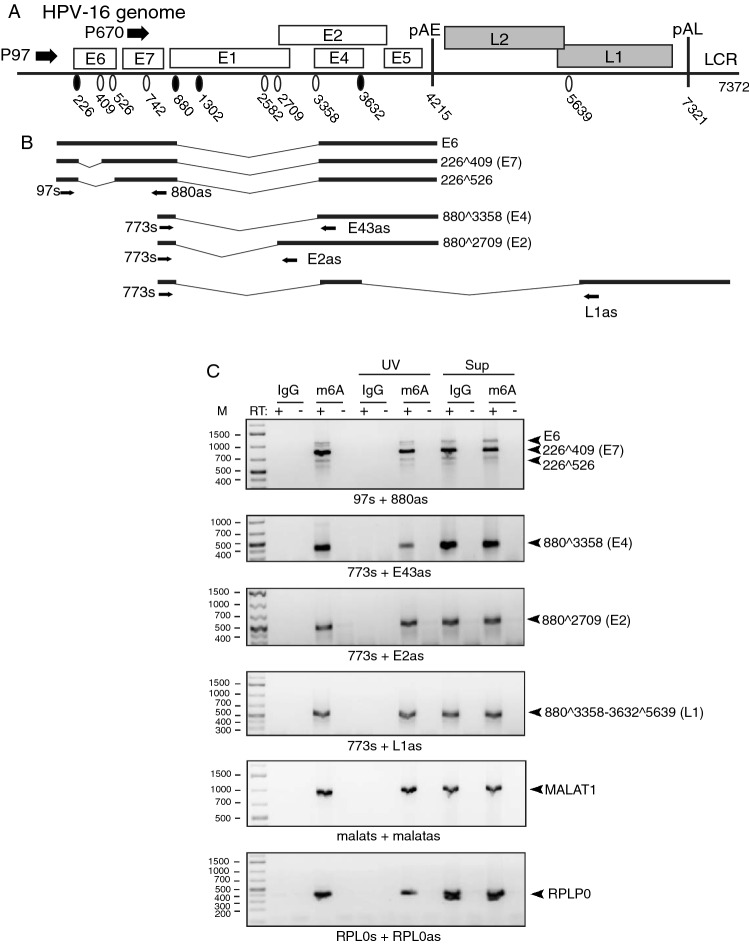
**A** Full-length HPV16 genome (numbers refer to the HPV16 reference strain GeneBank: K02718.1). Early and late genes are indicated. P97: HPV16 early promoter. P670: HPV16 late promoter. Black oval: splice donor. White oval: splice acceptor. pAE: HPV16 early polyadenylation site. pAL: HPV16 late polyadenylation site. LCR: HPV16 long control region. **B** Schematic representations of a subset of HPV16 alternatively spliced mRNAs produced from the HPV16 genome. Arrows indicate positions of RT-PCR primers. **C** RNA was extracted from HN26 cells and subjected to immunoprecipitation with either IgG or anti-m6A antibody (m6A) followed by RT-PCR on RNA extracted from the immunoprecipitated antibody–RNA complexes. HPV16 RT-PCR primers are indicated. RT-PCR primers used for amplification of HPV16 mRNAs, cellular RPLP0 mRNAs, and cellular MALAT RNAs are listed in Supplementary Table 1. The spliced HPV16 mRNAs represented by the various DNA fragments are indicated to the right. UV, immunoprecipitation samples treated with UV light prior to washing of the antibody-RNA complexes. Sup, RT-PCR on the supernatants obtained after immunoprecipitation of the HPV16 mRNAs. +  reverse transcription in the presence of reverse transcriptase (RT); − reverse transcription in the absence of RT

Finally, we wished to determine if HPV16 mRNAs are m6A-methylated in HPV16-driven cancer cells. We therefore extracted RNA from the HPV16-positive tonsillar cancer cell line HN26 [34]. This cell line was recently isolated from a tumor of a 48-year-old nonsmoking man with non-keratinizing, HPV16-positive tonsil oral squamous cell carcinoma, stage T2N0M0. The HN26 cells have an intact p53 gene and contain a complete episomal HPV16 DNA genome (Fig. 6A). A selected set of the alternatively spliced HPV16 mRNAs produced in this cell line is depicted (Fig. 6B). We subjected RNA extracted from the HN26 cells to immunoprecipitation with m6A-specific monoclonal antibody. RT-PCR on RNA extracted from these immunoprecipitations revealed that mRNAs encoding HPV16 E2, E4, E6, and E7 were immunoprecipitated by the m6A antibody, but not by IgG (Fig. 6C). Antibody-RNA complexes were also stabilized by UV cross linking prior to addition of protein G beads, with similar results (Fig. 6C). The hypermethylated MALAT1 lncRNA was also readily detected by the m6A antibody (Fig. 6C) as was the mRNA of the RPLP0 house keeping gene (Fig. 6C). Taken together, our results confirmed that HPV16 mRNAs are m6A-methylated in cancer cells which supported the idea that m6A-methylation plays an important role in the control of HPV16 gene expression.

## Discussion

Adenosines are more likely to be m6A-methylated if located in the so called DRACH motif, i.e., (A/G)AC(ACU). However, RNA-sequencing data have identified m6A modifications on other adenosines as well and has allowed the development of prediction servers for m6A sites, such as SRAMP [39]. We analyzed the HPV16 genome for the presence of potential m6A sites using SRAMP. Unlike the general pattern of m6A on cellular mRNAs, which clusters around start codons and in the 3’-UTR [19, 40], m6A on HPV16 mRNAs are predicted to cluster around HPV16 splice sites (Supplementary Fig. S3). The vast majority of all predicted m6A-methylated adenosine residues clustered downstream of the major HPV16 E6/E7 splice site SA409 and downstream of the HPV16 E2 splice site SA2709 (Supplementary Fig. S3). A few sites were predicted to be present in the small HPV16 exon located between splice sites SA3358 and SD3632 as well (Supplementary Fig. S3). Three predicted m6A sites immediately downstream of SA3358 coincide with a previously identified splicing enhancer, suggesting that m6A may modulate SA3358 through this splicing enhancer [41]. Interestingly, these are the HPV16 splice sites that were affected by the overexpression of ALKBH5, METTL3, and/or YTHDC1. In particular, overexpression of the ALKBH5 “eraser” inhibited splicing of the E6/E7 mRNAs and so did overexpression of the m6A “reader” YTHDC1. To reconcile these results, one may speculate that ALKBH5 removed m6A-adenosines thereby reducing the ability of a yet undisclosed cellular “reader” and splicing factor to bind to the mRNAs and enhance splicing, while YTHDC1 may bind to m6A sites on the same mRNAs and prevent the said cellular splicing factor from binding. In addition to these observations, METTL3 overexpression inhibited splicing to SA2709 and the production of E2 mRNAs, while at the same time promoting retention of the E1-encoding intron and production of the E1 mRNAs. These results were obtained also with the HPV16 pBEL reporter plasmid on which the E6/E7 region is absent, suggesting that m6A modifications in the E1/E2 region controlled splicing in the E1/E2 region, i.e., HPV16 3’-splice site SA2709. However, it remains to be determined if these predicted m6A sites are indeed methylated in cells and how this methylation potentially affects binding of cellular splicing factors to the HPV16 mRNAs.

The combined functions of the high-risk papillomavirus E6 and E7 proteins are required for the establishment of a productive viral infection and for transformation of HPV16-infected cells to cancer cells or for efficient immortalization of primary keratinocytes in vitro [42, 43]. E6 and E7 are both expressed in cancers that are caused by HPV16, i.e., various anogenital cancers, primarily cervical cancer, and head and neck cancer primarily tonsillar cancer. While E7 targets the cell cycle, E6 prevents apoptotic cell death. Therefore, a balanced expression of E6 and E7 is pivotal. The E7 mRNA (226^409^) is believed to be the major E7 producing mRNA while the unspliced mRNA mainly produces E6 [44, 45]. It is therefore of paramount importance for HPV16 to maintain a well-balanced splicing efficiency to allow for production of sufficient quantities of both E6 and E7. Control of E6/E7 mRNA splicing is crucial for HPV16 replication but also for induction and maintenance of cancers driven by HPV16. It has been shown that EGF-signaling can regulate the balance between unspliced E6 and spliced E7 mRNAs (226^409^) in HPV16-infected cells, with EGF-signaling promoting production of unspliced E6 mRNAs [46]. Recently, METTL3 was shown to have a positive effect on EGFR levels in cancer cells through translational regulation [47]. On the other hand, our results demonstrated that overexpression of the demethylase ALKBH5 increased the levels of unspliced E6 mRNA whereas METLL3 did not, which suggested that a reduction of m6A modifications inhibited E6/E7 mRNA splicing. It remains to be determined how METTL3 and ALKBH5 are involved in the control of HPV16 E6/E7 mRNA splicing in HPV16-infected cells.

Unexpectedly, overexpression of the reader YTHDC1 had a similar effect on HPV16 E6/E7 splicing as the eraser ALKBH5—it caused retention of the E6-encoding intron and promoted production of E6 mRNAs. Our results demonstrated that YTHDC1 physically associated with HPV16 mRNAs by the use of CLIP assay. These results may therefore suggest that YTHDC1 either interacts preferentially with m6A-containing mRNAs to prevent factors with a splicing enhancing function to bind to HPV16 mRNAs that YTHDC1 interacts with the HPV16 E6/E7 mRNAs and inhibit adjacent splicing factors or that YTHDC1 recruits factors with splicing inhibitory function. It appears that YTHDC1 can regulate splicing by recruiting various splicing factors. This has been described for a subset of cellular mRNAs to which YTHDC1 recruited SRSF3 to cause exon inclusion while blocking access to SRSF10 that promoted exon exclusion [24]. YTHDC1 was found to interact with SRSF3 also during mouse oocyte development together with SRSF7 and polyadenylation factor CPSF3, affecting both alternative splicing and alternative polyadenylation [48]. Interestingly, SRFS3 has been shown to control HPV16 mRNA splicing [49]. These results indicate that YTHDC1 can affect splicing in discrete ways. It remains to be determined how YTHDC1 promotes intron retention of HPV16 E6/E7 mRNAs.

The binding to RNA of other RNA-binding proteins than YTHDC1 may also be affected by m6A-methylation, for example members of the hnRNP-family of splicing factors. Some hnRNPs may be direct readers of m6A [19], e.g., hnRNPA2B1 that has been functionally linked to m6A-mediated splicing [25]. Furthermore, the binding of HuR [18], hnRNPG, and hnRNPC [20, 21] to regulatory RNA elements is altered in response to distortions of RNA- secondary structures by the presence of m6A modifications. All three proteins have previously been shown to bind RNA elements on HPV16 mRNAs and regulate HPV16 mRNA splicing [37, 38, 50, 51] and may therefore potentially be affected by m6A-methylation of HPV16 mRNAs. Recently, a splicing silencer RNA element located in the HPV16 E7-coding region has been identified. It acts by reducing production of the spliced E7 mRNA (226^409^) [38]. A similar sequence is present in HPV18 [49]. These RNA elements interacted with hnRNP A1, but it is currently unknown if the interactions between hnRNP A1 and the HPV16 RNA silencers are affected by m6A-methylation or by YTHDC1. Taken together, our results suggest that m6A-methylation of HPV16 mRNAs may function as an additional layer in the control of HPV16 mRNA splicing.

## Limitations of the study

These experiments do not allow us to conclude whether the effect on HPV16 mRNA splicing is a direct effect due to m6A-methylation of the HPV16 mRNAs or an indirect effect of m6A-methylation of cellular transcripts, e.g., those encoding splicing regulatory factors. In this respect, it may be of interest to note that high confidence m6A sites are present on mRNAs encoding SRSF2, a protein that may control HPV16 mRNA splicing. Since we have not mapped the exact sites of m6A-methylation on the HPV16 mRNAs, we do not know if HPV16 mRNAs produced from integrated HPV16 genomes found in the majority of the cancers caused by HPV16 are m6A-methylated.

## Supplementary Information

Below is the link to the electronic supplementary material.

Supplementary file1 (PDF 2364 KB)
