# Supplementary material for: Overexpression of m6A-factors METTL3, ALKBH5, and YTHDC1 alters HPV16 mRNA splicing
Source: Virus Genes. 2022 Feb 21;58(2):98–112. doi: 10.1007/s11262-022-01889-6 (PMC8948141; doi:10.1007/s11262-022-01889-6)
Supplement: Supplementary file 1 — Supplementary file1 (PDF 2364 KB) [file 11262_2022_1889_MOESM1_ESM.pdf]

SUPPLEMENTARY FIGURE LEGENDS

Supplementary Figure S1 Sequence of in vitro synthesized, m6A-containing RNA.

Supplementary Figure S2 (A) Left panel: Western blot with anti-Flag antibody on proteins harvested from HeLa cells transfected with empty pUC plasmid (-), pYTHDC1, pMETTL3, pMETTL14, pWTAP or pFTO. Middle panel: Western blot with anti-ALKBH5 antibody on proteins harvested from HeLa cells transfected with empty pUC plasmid (-) or pALKBH5. Right panel: Long exposure of Western blot with anti-Flag antibody on proteins harvested from HeLa cells transfected in duplicates with empty pUC plasmid (-), pMETTL3, pMETTL14 or pWTAP. **(B)** Effect of METTL3, METTL14, WTAP or all three combined on HPV16 E6/E7 mRNA splicing was monitored by RT-PCR with indicated primer pair on RNA extracted from HeLa cells transfected with pC97ELsLuc and empty pUC plasmid (-), pMETTL3, pMETTL14, pWTAP or pMETTL3, pMETTL14 and pWTAP combined. **(C)** Effect of METTL3, METTL14, WTAP or all three combined on HPV16 E2 mRNA splicing was monitored by RT-PCR with indicated primer pair on RNA extracted from HeLa cells transfected with pC97ELsLuc and empty pUC plasmid (-), pMETTL3, pMETTL14, pWTAP or pMETTL3, pMETTL14 and pWTAP combined. **(D)** Effect of FTO and ALKBH5 overexpression on HPV16 L1- and E4- mRNA splicing was monitored by RT-PCR with indicated primer pairs on RNA extracted from pBELsLuc with empty pUC plasmid (-), pFTO or pALKBH5.

Supplementary Figure S3 (**A**) m6A site prediction on the HPV16 genome performed using the online software SRAMP *(http://www.cuilab.cn/sramp/)* (Zhou). Selected HPV16 splice sites SD226, SA409, SD880, SA2709, SA3358 and SD3632 are indicated. Numbers refer to the HPV16 reference strain (GeneBank: K02718.1). HPV16 early and late polyadenylation signals pAE and pAL, respectively, are indicated. **(B)** A subset of alternatively spliced HPV16 mRNAs are depicted.

Supplementary Figure S1. *In Vitro transcribed cloning vector RNA sequence.*

AGGATTTCGAGCGTGGGTCACTGAGCTCAGTGGACGAAAACTCACGTTAAGGGATTTT
GGTCATGAGATTATCAAAAAGGATCTTCACCTAGATCCTTTTAAATTAAAAATGAAGTTTT
AAATCAATCTAAAGTATATATGAGTAAACTTGGTCTGACAGTTACCAATGCTTAATCAGT
GAGGCACCTATCTCAGCGATCTGTCTATTTCGTTCATCCATAGTTGCCTGACTCCCCGT
CGTGTAGATAACTACGATACGGGAGGGCTTACCATCTGGCCCCAGTGCTGCAATGATA
CCGCGAGACCCACGCTCACCGGCTCCAGATTTATCAGCAATAAACCAGCCAGCCGGAA
GGGCCGAGCGCAGAAGTGGTCCTGCAACTTTATCCGCCTCCATCCAGTCTATTAATTGT
TGCCGGGAAGCTAGAGTAAGTAGTTCGCCAGTTAATAGTTTGCGCAACGTTGTTGCCAT
TGCTACAGGCATCGTGGTGTCACGCTCGTCGTTTGGTATGGCTTCATTCAGCTCCGGTT
CCCAACGATCAAGGCGAGTTACATGATCCCCCATGTTGTGCAAAAAAGCGGTTAGCTCT
TTCGGTCCTCCGATCGTTGTCAGAAGTAAGTTGGCCGCAGTGTTATCACTCATGGTTAT
GGCAGCACTGCATAATTCTCTTACTGTCATGCCATCCGTAAGATGCTTTTCTGTGACTG
GTGAGTACTCAACCAAGTCATTCTGAGAATAGTGTATGCGGCGACCGAGTTGCTCTTGC
CCGGCGTCAATACGGGATAATACCGCGCCACATAGCAGAACTTTAAAAGTGCTCATCAT
TGGAAAACGTTCTTCGGGACGAAAACTCTCAAGGATCTTACCGCTGTTGAGATCCAGTT
CGATGTAACCCACTCGTGCACCCAACTGATCTTCAGCATCTTTTACTTTCACCAGCGTTT
CTGGGTGAGCAAAAACAGGAAGGCAAAATGCCGCAAAAAAGGGAATAAGGGCGACA
CGGAAATGTTGAATACTCATACTCTTCCTTTTTCAATATTATTGAAGCATTTATCAGGGTT
ATTGTCTCATGAGCGGATACATATTTGAATGTATTTAGAAAAATAACAAATAGGGGTTCC
GCCGCAATTTTCCCCAAAAATGCCACCTGAACTTCAAGAAAC

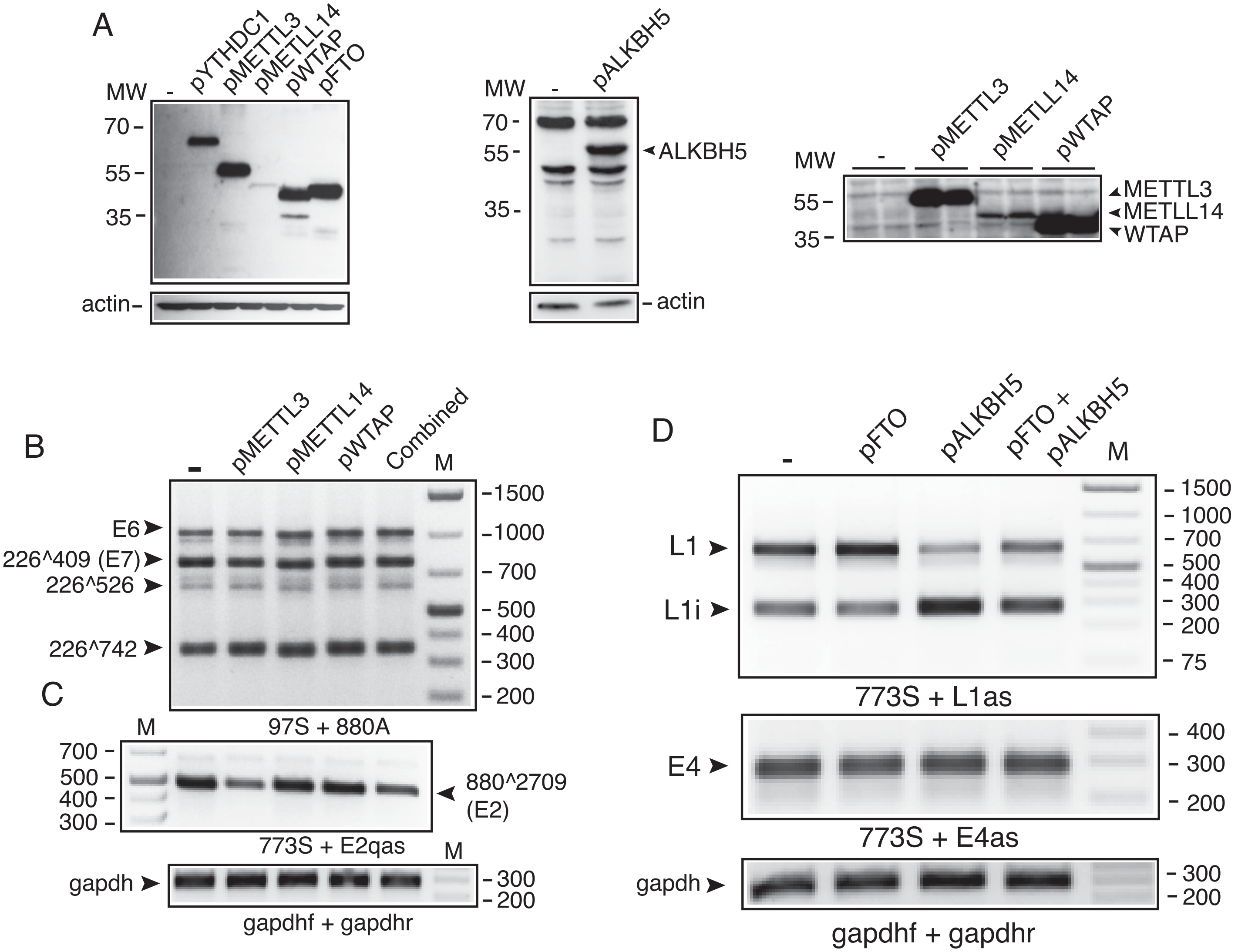
A
MW
-
pYTHDC1
pMETTL3
pMETLL14
pWTAP
pFTO
70
55
35
actin
pALKBH5
ALKBH5
pMETLL14
METTL3
METLL14
WTAP
B
pMETTL3
pMETTL14
pWTAP
Combined
M
1500
1000
700
500
400
300
200
E6
226^409 (E7)
226^526
226^742
C
97S + 880A
880^2709
(E2)
773S + E2qas
gapdh
gapdhf + gapdhr
D
pFTO
pALKBH5
pFTO +
pALKBH5
L1
L1i
75
773S + L1as
E4
773S + E4as

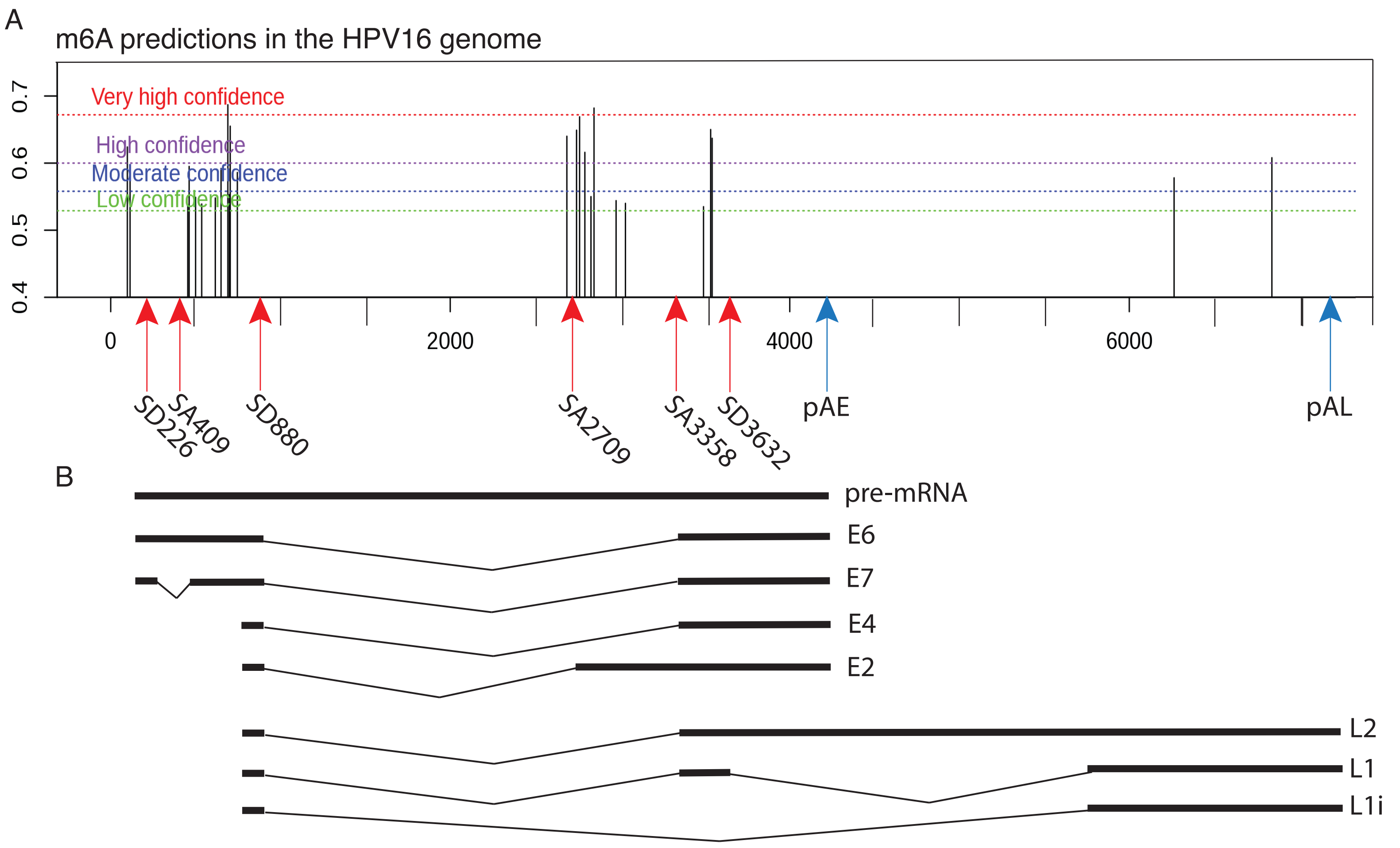
A
m6A predictions in the HPV16 genome
Very high confidence
High confidence
Moderate confidence
Low confidence
0.4
0.5
0.6
0.7
0
2000
4000
6000
SD226
SA409
SD880
SA2709
SA3358
SD3632
pAE
pAL
B
pre-mRNA
E6
E7
E4
E2
L2
L1
L1i

Supplementary Table 1. Primer list.

RT-PCR primer name	Amplified sequences	Sequence
97s	HPV16	GTCGACCTGCAATGTTTCAGGACCC
880as	HPV16	GAAACCATAATCTACCATGGCTGATC
X556A	HPV16	GCTCGAGCAGCTGGGTTTCTCTACGTGTT
773s	HPV16	GCACACACGTAGACATTCGTACTTTG
E4as	HPV16	TGCTGCCTAATAGTTTCAGGAGAGG
E43as	HPV16	GTGGTGTGGCAGGGGTTTCCGGTGTC
E42as	HPV16	CGGTGCCCAAGGCGACGGCTTTGG
L1as	HPV16	GCAACATATTCATCCGTGCTTACAACC
E2as	HPV16	CAGCCAGCGTTGGCACCACCT
E2qas	HPV16	CAGCCAGCGTTGGCACCACCT
16S	HPV16	TATGTATGGTATAATAAACACGTGTGTATGTG
16A	HPV16	GCAGTGCAGGTCAGGAAAACAGGGATTTGGC
MALAT1s	MALAT1	CGTAGACCAGAACCAATTTAGAAG
MALAT1as	MALAT1	CATATTGCCGACCTCACGGAT
MALAT1asQ	MALAT1	AGCACCTGGGTCAGCTGTCAAT
RPL0s	RPLP0	ACCTGGAAGTCCAACTACTTCCTTA
RPL0as	RPLP0	GATCTCAGTGAGGTCCTCCTTG
gapdhf	GAPDH	ACCCAGAAGACTGTGGATGG
gapdhr	GAPDH	TTCTAGACGGCAGGTCAGGT
T7s	*In vitro* RNA	ACGTTAAGGGATTTTGGTCATGAGA
T7as	*In vitro* RNA	TCAAATATGTATCCGCTCATGAGA
